# Cross-Cultural Adaptation and Validation of the Emotional Inhibition Scale in a Chinese Cancer Sample

**DOI:** 10.3389/fpsyg.2021.654777

**Published:** 2021-09-03

**Authors:** Liping Liu, Yikai Xu, Yanni Wu, Xiaoxia Li, Chunlan Zhou

**Affiliations:** ^1^Department of Medical Imaging Center, Nanfang Hospital, Southern Medical University, Guangzhou, China; ^2^Department of Nursing, Nanfang Hospital, Southern Medical University, Guangzhou, China; ^3^Department of Plastic and Cosmetic Surgery, Nanfang Hospital, Southern Medical University, Guangzhou, China

**Keywords:** cancer, reliability, validity, emotional inhibition, Chinese, confirmatory factor analysis

## Abstract

The Emotional Inhibition Scale (EIS) is a brief measure based on a four-factor model with documented validity in a mood disorder sample that may be useful for assessing emotional inhibition in patient populations, such as individuals with cancer. The present study adapted the EIS to Chinese conditions and examined the psychometric characteristics of the EIS in patients with cancer. The recruited participants comprised a sample of 100 patients (sample 1) and a sample of 202 patients (sample 2) with cancer. The two samples (sample 1 and sample 2) with cancer completed surveys including the EIS. The Toronto Alexithymia Scale-20 was completed by the two sample groups to assess criterion validity. Statistical analyses included internal consistency (sample 1), exploratory factor analyses (EFAs; sample 1), and confirmatory factor analyses (CFAs; sample 2). The results showed that EFA and CFA confirmed the four-factor solution proposed by the original authors (verbal inhibition, self-control, disguise of feelings, and timidity). The internal consistency and test-retest reliability of the EIS were satisfactory. In conclusion, the EIS demonstrated acceptable reliability and validity for assessing emotional inhibition in Chinese-speaking patients with cancer and may be a useful measure for assessing the level of emotional inhibition and the effect of emotional disclosure interventions.

## Introduction

Emotional inhibition (EI) refers to the tendency to consciously inhibit emotional expressions while emotionally aroused (Coggins and Fox, 2009; Ellis and Cromby, 2012; Traue et al., 2016). Overt EI is characterized by unemotional language, reduced expressiveness, and shyness, all of which are linked to dysfunctional bodily reactions and may be adaptive in a short-term social stress situation. EI in the long term is considered an underlying cause of psychopathology and may adversely impact psychological and health outcomes (Traue et al., 2016). However, little is known about the correlates and potential causes of EI. EI has been linked to the adaptation and well-being of patients. Recent studies strongly related EI to anxiety disorders (Zimmermann et al., 2015; Peh et al., 2017), depression (Langner et al., 2012; Li et al., 2015), substance misuse (Marceau et al., 2018), eating disorders (Ferrer et al., 2017), paranoia (Nittel et al., 2018), and borderline personality disorder (Popolo et al., 2014; Salvatore et al., 2016).

Patients with cancer who reported the use of generally less adaptive strategies to regulate or express their emotions (e.g., suppression or inhibition) also reported more emotional distress and lower well-being (Peh et al., 2016, 2017). A wide range of self-report measures have been developed to assess the emotional regulation and related constructs (e.g., the Emotion Regulation Questionnaire; the Cognitive Emotion Regulation Questionnaire; and the Emotional Expressivity Scale). However, decisions regarding which measure to use are challenging given the diverse conceptualizations and elements of emotional regulation (Brandão et al., 2016). Most instruments focus on tendencies to suppress the expression of negative emotions and include a wide range of specific strategies, including conscious suppression and increasing defensive strategies, which help individuals to eliminate negative affect (Brandão et al., 2016). The results are used to identify patients who might be at risk for emotional disorders and might benefit from supportive interventions. However, these assessments mostly focus on general measures to assess the ability of coping strategies to regulate emotions or emotional expression, which indirectly assess the inhibition of emotions (Brandão et al., 2016). Few assessments have directly examined the trait qualities of EI. In summary, the lack of assessment tools has limited the development of EI research in China.

Robert Kellner developed the Emotional Inhibition Scale (EIS) in 1986 as a self-rating instrument based on clinometric principles that are used to evaluate the beliefs of a person in suppressing feelings and emotions (Kellner, 1986; Grandi et al., 2011). The EIS is the only reliable instrument that explains the behavior and intrinsic features of EI. In recent studies, the EIS has been administered to cardiac recipients, hirsute women, patients with hypochondriacal attitudes, patients with personality disorders, patients with somatic concerns, and patients with panic disorders (Fava et al., 1989; Grandi et al., 2011; Salvatore et al., 2016; Dimaggio et al., 2018). The EI plays an important role in the onset, evolution, and outcome of psychological difficulties (Soto et al., 2011; Ellis and Cromby, 2012). The psychometric attribute of the EIS should be tested to further develop its incremental validity for EI and its sensitivity to change after treatments designed to increase emotional disclosure.

The present study was undertaken to fill the gap mentioned above. This study was designed to (1) translate the EIS into simplified Chinese and provide cross-cultural adaptation in China, (2) evaluate the validity and reliability of the Chinese version of the EIS (C-EIS) in a sample of Chinese-speaking patients with cancer, and (3) determine the demographics affecting the EI of patients with cancer. The current findings contribute to a better understanding of EI in Chinese patients with cancer to help medical staff develop specialized psychological interventions for these patients in the future. This scale may be a useful measure to assess the level of EI and the effects of emotional disclosure interventions.

## Materials and Methods

### Translation and Modification Procedure

The permission to translate and use the EIS was obtained *via* email correspondence with the author of the original version (Kellner, 1986). The translation process and cultural adaptation were based on the suggestions of classic Brislin's double translation and back translation guidelines (Cha et al., 2007). Using the same setup as the original English language version, two researchers with knowledge in psychology and extensive experience in translating psychological tests and measures translated the EIS from English to Chinese, and two bilingual research assistants translated it back to English. A panel of six experts was convened to discuss the appropriateness of the translations item by item and determine the cultural equivalence of the C-EIS. A pilot test consisting of 30 patients with cancer was implemented with the prefinal version of the C-EIS, and further alterations to phrasing were performed according to the feedback from participants on the scale. For example, item 9 “Do you speak up for your rights?” was revised to “Do you stick to your rights.” Item 15, “Would you like to tell someone how you feel but are too inhibited to do so,” was revised to “Would you like to tell someone how you feel but it is too hard for you to do so.” After that, all the items were rediscussed and adjusted by the expert panel until there were no substantial differences, and the final version of the C-EIS was formed.

### Participants and Procedures

A cross-sectional study design was used to assess the psychometric properties of the C-EIS. The data were collected at a tertiary grade A comprehensive hospital in Guangzhou, China, between January and April 2019. Using convenience sampling, we invited patients to participate in the study if they (1) had a confirmed pathological cancer diagnosis, (2) were over 18 years old, (3) could answer the questionnaire independently, and (4) provided written informed consent. Patients were excluded if they had a diagnosed psychiatric disorder. To better represent the cancer population, this study adopted sampling of participants with cancer, and the incidence rates ranked in the top 10 according to the latest global cancer data of 2018, which are listed as follows: lung cancer, breast cancer, prostate cancer, colon cancer, nasopharyngeal cancer, gastric cancer, liver cancer, rectal cancer, esophageal cancer, and uterine cervical cancer.

A sample size should be 5–10 times larger than the number of items in the scale based on the Kendall sample estimation method (Zou, 2012). A minimum sample size of 80 was determined because the number of items in the EIS was 16. This survey was performed as a two-phase process. First, 100 patients took the survey, which exceeded the required sample size mentioned above. However, because the factors determined by exploratory factor analysis (EFA) were slightly different from the original English version, we involved another group of subjects for confirmatory factor analysis (CFA) because the data for EFA could not be used repeatedly for CFA. Another group of 202 patients was involved in the second phase, which exceeded the suggested minimum sample size of 200 for a CFA (Marsh et al., 1998). Finally, 30 patients who were randomly selected from the two samples completed the C-EIS again after a 3-week interval to evaluate test–retest reliability.

This study was performed based on the principles outlined in the Declaration of Helsinki 2013 (Mastroleo, 2016), and approval was obtained from the Medical Ethics Committee of the case hospital (NFEC-2018-049). Informed consent was signed by all participants, and the survey questionnaires were completed voluntarily. It was emphasized that the privacy of participants was kept strictly confidential throughout the whole study process.

### Measurements

#### Sociodemographic and Clinical Characteristics

Sociodemographic data, including age, sex, marital status, faith, family income, education, and profession, were collected. Clinical characteristics data on diagnosis, disease information, medical expenses payment method, and recent treatment were also collected.

Emotional Inhibition Scale

The EIS was developed by Robert Kellner (Kellner, 1986) to measure the belief of a person in suppressing the feelings and emotions found in psychosomatic investigations. The EIS includes four subscales: timidity, verbal inhibition, self-control, and disguise of feelings. It is a 16-item instrument rated on a 5-point Likert-type scale (no = 0, always = 4). The sum of the 16 items is the total EI score, which ranges from 0 to 64. Higher scores demonstrate a higher degree of introversion, emotional restriction, and timidity. The EIS has good construct validity and internal reliability (Grandi et al., 2011). The Cronbach's α coefficient for the scale was 0.95, and the coefficients for the four subscales ranged from 0.77 to 0.95. The CFA results showed acceptable global goodness of fit [comparative fit index (CFI) = 0.925].

#### Toronto Alexithymia Scale-20

The Toronto Alexithymia Scale (TAS)-20 was conceived by Bagby et al. (1994) to measure the difficulty of a person in identifying, describing, and communicating one's feelings to others. The concept of alexithymia is related to EI. The TAS-20 is a 20-item instrument rated on a 5-point Likert-type scale (1 = strongly disagree, 5 = strongly agree). This instrument is composed of three subscales: externally oriented thinking, difficulty in describing feelings, and difficulty in identifying feelings. The Chinese version of the TAS-20 was used in this study, and it has good construct validity and internal reliability (Ling et al., 2016). The Cronbach's α coefficient for the scale was 0.87, the coefficients for the four subscales ranged from 0.47 to 0.84, and the CFA results showed acceptable global goodness of fit [CFI = 0.92, goodness-of-fit index (GFI) = 0.94].

### Statistical Procedures

Statistical Package for the Social Sciences (SPSS) 17.0 software was used to perform EFA and descriptive statistics that summarized the sociodemographic and clinical characteristics of all the participants. Automated Meteorological Observation Station (AMOS) 24.0 software was used to perform CFA. *P* < 0.05 was considered statistically significant.

Three types of coefficients were used to test the reliability of the C-EIS. Cronbach's α coefficient was calculated to examine internal consistency reliability, where α coefficients > 0.7 indicated that the reliability coefficient was acceptable. The Spearman-Brown coefficient was used to test the split-half reliability of the scale, and the Pearson correlation coefficient r between the scores of the test-retests was used to explore the total test–retest reliability (*r* > 0.8 showed good test-retest reliability). The intraclass correlation coefficient (ICC) is also a good indication for test-retest reliability. A recent study provided ICC values that ranged from fair agreement (<0.40) to almost perfect agreement (>0.80). The corrected item-total correlation was used as a type of item analysis, with a value >0.30, as recommended (Andresen, 2000).

Content validity for the scale may be measured by using the content validity index (CVI). An expert panel (four clinical nursing specialists, one nursing magazine editor, and one nursing educator) was formed to assess the CVI of the C-EIS. An evaluation scale was distributed to the expert panelists. The experts were asked to correspondingly rate the relevance and clarity of each item using 4-point Likert scales ranging from 1 (very unclear and needs full revisions) to 4 (very clear and does not need to be revised). The CVI was calculated for the scale level (S-CVI) and each item level (I-CVI), with a minimum acceptable value of 0.78, as recommended (Lynn, 1986).

Construct validity was estimated using item analysis, EFA, and CFA. EFA was used for dimension reduction and identification of the factor structure (principal components with varimax rotation). The following criteria were used for item retention and factor extraction: (a) each factor had three items loading or above; (b) factor loading >0.40; (c) eigenvalue >1.0; and (d) no cross-loading items on two factors or above (Fang, 2001). The EFA-derived structure was investigated using Velicer's minimum average partial (MAP) test combined with parallel analysis to corroborate the number of EIS factors (Ye et al., 2018).

The factorial structure of the C-EIS was tested using the CFA model identified in the exploratory study. The parameter estimates (factor loadings and covariances) and model fit indices were used to test the model goodness of fit. The following criteria of model fit were used: standardized root mean square residual (SRMR) < 0.08; GFI > 0.9; ratio of the chi-square statistic to degrees of freedom (χ^2^/df) < 3; Tucker–Lewis index (TLI) > 0.9; root mean square error of approximation (RMSEA) < 0.08, and CFI > 0.9 (Marsh et al., 1998).

Construct validity was also assessed by comparisons of contrasted groups based on the expected hypotheses of differences in C-EIS scores for groups split by different disease staging, type of cancer, and time since diagnosis. The following hypotheses were proposed: (1) Patients who have more severe cancer will have higher scores because pain from cancer may aggravate the inhibition of emotion (Cardenal et al., 2012), (2) The type and site of cancer may impact the emotional expression of patients because various cancers may have entirely different psychosocial factors associated with them (Batty et al., 2017), and (3) Patients who have been living with cancer for a longer time may have higher EI scores. The diagnosis and treatment of cancer is a source of distress, and EI may be more obvious with more time to understand the situation (Chapman et al., 2013).

Convergent validity was assessed using the correlation between the scores of the C-EIS and those of the TAS-20. Normality analysis was performed before Pearson analysis. This study considered 0.30 as the minimum acceptable Pearson correlation value (Heinl et al., 2016).

## Results

In the first phase of the 107 survey questionnaires distributed to the participants, two were missed and five were excluded due to incomplete items. Therefore, a total of 100 participants were included, with a valid response rate of 93.45%. In the second phase of the 212 survey questionnaires distributed to the participants, four were missed and six were excluded due to incomplete items. Finally, a total of 202 participants were included, with a valid response rate of 95.28%. The response rate of test–retest was 100%.

### Sociodemographic and Clinical Characteristics

A total of 302 patients were involved in the two-phase process. Sample 1 was comprised of 27 women and 73 men (mean age, 58.1; SD, 10.9 years; range 22–77 years), and sample 2 was comprised of 97 women and 105 men (mean age, 51.7; SD, 12.5 years; range 20–81 years). Nearly one-quarter of the sample 1 group had nasopharyngeal cancer, and one-quarter of the sample 2 group had breast cancer. There were some common characteristics of the two samples. Nearly half of the diseases were stage II, and most treatments were surgery and chemotherapy. Nearly half of the household monthly income was ≤ 3,500 Chinese yuan, and medical expenses were paid through social security. Nearly 70% of the time since diagnosis was <1 year (Table 1). For the significance level of demographic factors on the EIS, only three significant variables were identified in the multivariate analysis (Supplement 1): sex (β = 0.115, adjusted *R*^2^ = 0.033, *p* = 0.048), place of residence (β = 0.118, adjusted *R*^2^ = 0.057, and *p* = 0.039), and disease stage (β = 0.358, adjusted *R*^2^ = 0.040, and *p* = 0.046).

**Table 1:** Sociodemographic and clinical characteristics.

**Characteristics**	**Value**	
	**Sample 1**	**Sample 2**
Age	58.1 ± 10.9 (*n* = 100)	51.7 ± 12.5 (*n* = 202)
**Gender (%)**		
Male	73 (73.0)	105 (52.0)
Female	27 (27.0)	97 (48.0)
**Religious belief (%)**		
Yes	28 (28.0)	40 (19.8)
No	72 (72.0)	162 (80.2)
**Place of residence**		
Town	54 (54.0)	102 (50.5)
Countryside	46 (46.0)	100 (49.5)
**Profession**		
Civil servant	16 (16.0)	32 (15.8)
Housewife	6 (16.0)	36 (17.8)
Businessman	9 (9.0)	14 (6.9)
Farmer	29 (29.0)	57 (38.2)
Worker	22 (22.0)	24 (11.9)
Other	18 (18.0)	39 (19.3)
**Marital status (%)**		
Married	93 (93.0)	186 (92.1)
Single/divorced/widowed	7 (7.0)	16 (7.9)
**Education level (%)**		
Primary or under	27 (27.0)	42 (20.8)
Junior high school	33 (33.0)	72 (35.6)
Senior high school	18 (18.0)	40 (19.8)
College or above	22 (22.0)	48 (23.8)
**Medical expenses payment method**		
New rural medical insurance	39 (3.09)	79 (39.1)
Social security	57 (57.0)	115 (56.9)
Self-pay	4 (4.0)	8 (4.0)
**Household monthly income (Chinese Yuan) (%)**		
<3,500	44 (44.0)	120 (59.4)
3,500–5,000	28 (28.0)	69 (34.2)
>5,000	28 (28.0)	13 (6.4)
**Duration of disease (years) (%)**		
<1	77 (77.0)	139 (68.8)
1–3	13 (13.0)	44 (21.8)
<3–5	5 (5.0)	11 (5.4)
>5	5 (5.0)	8 (4.0)
**Types of cancer**		
Lung cancer	14 (14.0)	42 (20.8)
Breast cancer	5 (5.0)	51 (25.2)
Prostate cancer	5 (5.0)	11 (5.4)
Colon cancer	13 (13.0)	16 (7.9)
Nasopharyngeal cancer	26 (26.0)	17 (8.4)
Gastric cancer	13 (13.0)	16 (7.9)
Liver cancer	4 (4.0)	19 (9.4)
Rectal cancer	11 (11.0)	8 (4.0)
Esophagus cancer	9 (9.0)	7 (3.5)
Uterine cervical cancer	0 (0.0)	15 (7.4)
**Whether to transfer**		
Yes	39 (39.0)	70 (34.7)
No	61 (61.0)	132 (65.3)
**Whether recurrence**		
Yes	13 (13.0)	26 (12.9)
No	87 (87.0)	176 (87.1)
**Disease staging**		
I stage	3 (3.0)	7 (3.5)
II stage	47 (47.0)	90 (44.6)
III stage	10 (10.0)	35 (17.3)
IV stage	40 (40.0)	70 (34.7)
**Recent treatment**		
Surgery	50 (50.0)	57 (28.2)
Chemotherapy	39 (39.0)	88 (43.6)
Radiotherapy	2 (2.0)	6 (3.0)
Targeted therapy	0 (0.0)	31 (15.3)
Nutritional treatment	1 (1.0)	8 (4.0)
Other	8 (8.0)	12 (5.9)

### Psychometric and Validation Testing

#### Reliability

Item analysis and reliability results are listed in Table 2. The Cronbach's α coefficient of the EIS was 0.717 for the total scale and 0.602–0.882 for the four subscales. For split-half reliability, the Spearman–Brown coefficient was 0.755 (*P* < 0.05). The total test–retest reliability was 0.855, which indicates great consistency between the two testing times. Table 3 lists the ICC values for the four domains. The item-total correlations ranged from 0.389 to 0.802.

**Table 2:** Mean, SD, item analysis, and reliability analysis of C-EIS (*n* = 100).

**Item number**	**Mean**	**SD**	**Corrected item-total correlation**	**α if item deleted**	**α**
**Verbal inhibition**					0.882
EIS2	1.99	1.096	0.738	0.863	
EIS4	1.91	1.102	0.776	0.829	
EIS5	1.77	1.024	0.803	0.807	
**Self-control**					0.749
EIS1	2.17	1.295	0.505	0.715	
EIS8	2.35	1.184	0.615	0.651	
EIS10	2.61	1.081	0.645	0.642	
EIS14	1.89	1.238	0.434	0.752	
**Timidity**					0.602
EIS3	1.92	1.316	0.389	0.533	
EIS6	1.63	1.315	0.453	0.436	
EIS7	2.07	1.281	0.389	0.532	
**Disguise of feeling**					0.784
EIS12	1.68	1.18	0.561	0.747	
EIS13	1.66	1.139	0.593	0.73	
EIS15	1.72	1.198	0.6	0.727	
EIS16	1.84	1.07	0.612	0.722	

**Table 3:** Reliability analysis of the C-EIS.

**Subscale**	**Mean (SD)**	**Cronbach alpha**	**Range ICC**	**Subscale ICC**	**Test mean (SD)**	**Retest mean (SD)**
Verbal inhibition	5.67 (2.90)	0.882	0.30–0.86	0.62	9.52 (2.62)	9.20 (3.19)
Self-control	9.02 (3.63)	0.749	0.46–0.94	0.811	12.73 (3.11)	13.67 (3.74)
Timidity	5.62 (2.92)	0.602	0.69–0.88	0.944	6.74 (3.00)	6.93 (2.92)
Disguise of feeling	6.90 (3.58)	0.784	0.10–0.62	0.477	10.40 (2.59)	11.07 (3.22)

*C-EIS, Chinese version of Emotional Inhibition Scale; ICC, intraclass correlation coefficient*.

#### Content Validity

The S-CVI value was 0.915, and the I-CVI value was not less than 0.83, which indicated good content validity of the C-EIS. The expert panel reduced the risk of errors from inherent differences in the language structure between Chinese and English and helped identify the most accurate and easily understood terms for the C-EIS.

### Exploratory Factor Analysis

EFA of the 14 items revealed a Kaiser–Meyer–Olkin value of 0.717, and the Bartlett spherical test value was 485.45 (χ^2^ = 485.5, df = 91, and *p* < 0.001), which indicated that it was adequate for EFA. First, five common factors, with an eigenvalue of 1.0 or greater, were confirmed by an EFA of the 16 items. The factor loading of item 9 (0.378) was too low to be retained. Item 11 was deleted because only one item loaded on one extra factor. Finally, a four-factor structure was extracted. The first four eigenvalues from the actual dataset of the EIS scores in the parallel analysis were 3.06, 1.86, 1.52, and 1.08. The corresponding first four 95th-percentile random-data eigenvalues were 1.46, 1.34, 1.27, and 1.21, respectively, which indicates that four factors were the best option for the structure of the EIS (Supplement 2). The MAP test showed that when the root was 3 or 4, we obtained the smallest average 4th-power partial correlation of 0.00375. A total of 65.6% of the variance in the data were explained, of which factor 1, 2, 3, and 4 explained 18.1, 17.8, 17.0, and 12.7% of the variance, respectively. The factor loadings for the 14 items ranged from 0.602 to 0.882, which satisfied the criterion of 0.4 or above (Table 4). The four factors were named based on the nature of the items loading on the corresponding factor: verbal inhibition (factor 1), self-control (factor 2), timidity (factor 3), and disguise of feeling (factor 4).

**Table 4:** The exploratory factor analysis results of the C-EIS (*n* = 100).

**Item number and description**	**Factors**			
	**1**	**2**	**3**	**4**
**Verbal inhibition**				
(5). Do you tell people exactly what you think?	**0.896**	0.045	−0.153	0.031
(4). Do you show how you feel?	**0.891**	0.001	−0.08	−0.183
(2). Do you find it easy to talk about your feelings?	**0.856**	0.121	−0.187	−0.03
**Disguise of feeling**				
(16). Do you let your friends see what your mood is?	0.172	**0.789**	−0.092	−0.014
(12). Do you stop yourself from saying something because it might hurt another person?	0.111	**0.776**	−0.01	−0.138
(15). Would you like to tell someone how you feel but are too inhibited to do so?	−0.004	**0.768**	0.147	0.073
(13). Do you feel that you let people take advantage of you?	−0.12	**0.765**	0.16	0.185
**Self-control**				
(10). Do you try to appear calm when you are anxious and worried?	−0.119	0.023	**0.831**	0.021
(8). When you are angry, do you try to control yourself?	−0.13	0.016	**0.817**	−0.013
(1). Do you try to be polite even when people are rude to you?	−0.231	0.16	**0.681**	−0.079
(14). Do you pretend to be cheerful even when you feel sad?	0.023	0.007	**0.646**	0.304
**Timidity**				
(6). Do you find it difficult to insist on your rights?	0.019	0.202	0.04	**0.752**
(3). Do you find it difficult to speak up when you feel that you are being wronged?	0.02	−0.021	−0.015	**0.724**
(7). Do you find it difficult to talk about your true feelings even with close friends?	−0.237	−0.101	0.117	**0.708**
% of the variance	18.05	17.81	17	12.75
Cumulative variance	18.05	35.86	52.83	65.58

*Factor loadings with an absolute value >0.400 are displayed in bold*.

### Confirmatory Factor Analysis

**Figure 1:**
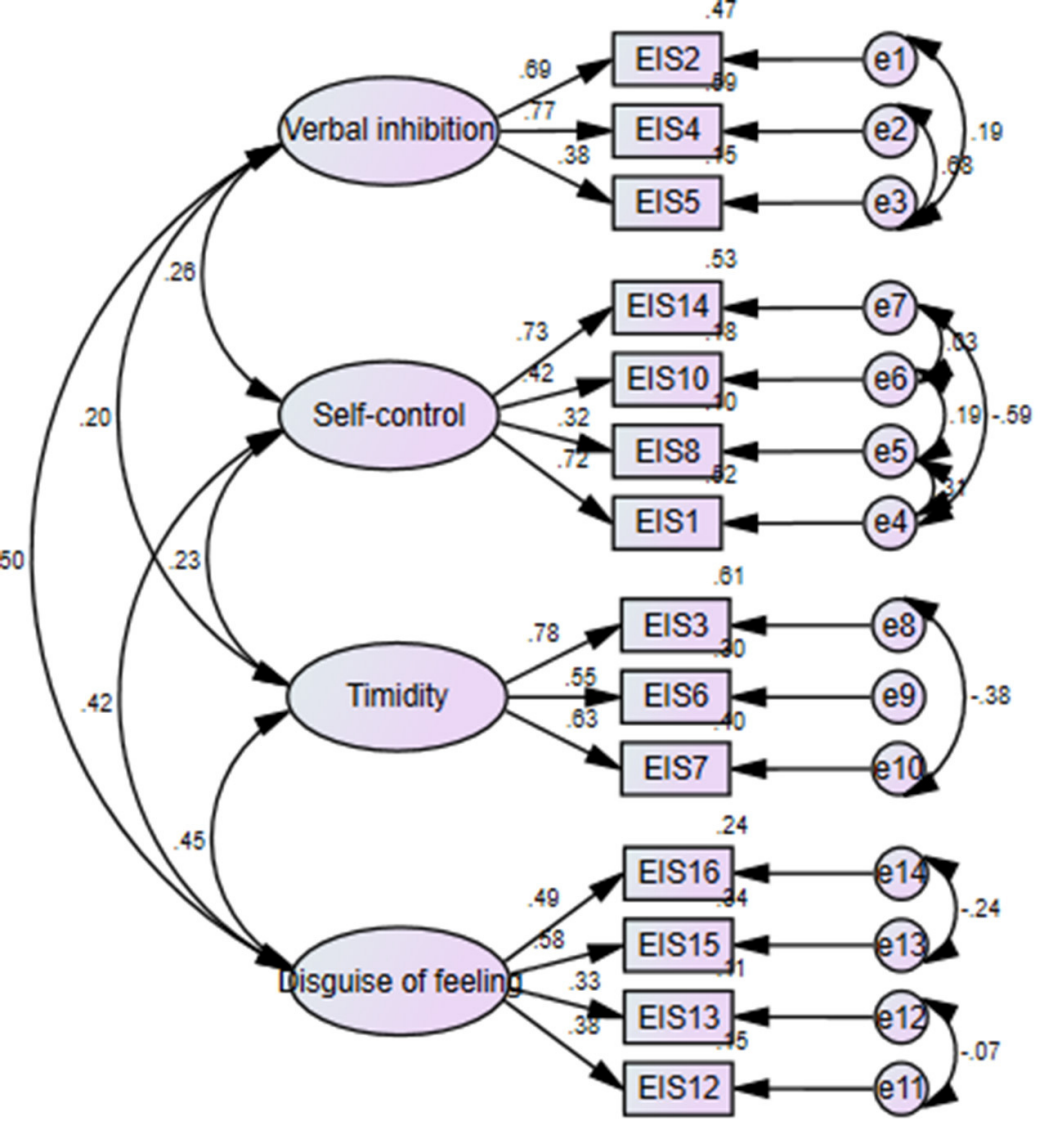
Confirmatory factor analysis.

The SEM confirmed the EFA-derived four-factor structure, and some of the results of the goodness of fit indices were χ^2^/df = 1.438 (=*p* < 0.001), RMSEA = 0.061, TLI = 0.861, and IFI = 0.911, which indicates that a satisfactory model fit the data in four-factor CFA (Table 5). The factor loading ranged from 0.32 to 0.83. Based on the modification indices, several paths of covariance between error and items were added to achieve an improved fitting model. The paths between observed variables, latent variables, and residuals are shown in Figure 1.

**Table 5:** Goodness-of-fit indices for the four-factor model in CFA (*n* = 202).

	**χ^2^/df**	**RMSEA**	**SRMR**	**CFI**	**TLI**	**IFI**	**GFI**
Result	1.741	0.061	0.086	0.905	0.861	0.911	0.929

The contrasting group comparisons showed that the hypothesis of expected differences for patients with esophageal cancer and more advanced cancer stage had higher scores for the C-EIS. However, there was no significant difference for time since cancer diagnosis (Table 6).

**Table 6:** Contrasted group comparisons (*n* = 302).

**Subgroups comparisons**	**Statistic value**	**Adjusted *P***
Cancer staging	4.221	0.004^*^
I–II stage	78.87	**0.035**
I–III stage	82.64	**0.040**
I–IV stage	99.82	**0.003**
II–III stage	3.77	1.00
II–III stage	20.95	0.362
II–IV stage	17.18	1.00
Types of cancer	1,838.87	<**0.01**^**#**^
Time since cancer diagnose (year)	2.285	0.319^*^

# *χ^2^-value;*

* *rank sum test*.

*P value < 0.05 are displayed in bold*.

### Convergent Validity

Although the strength of associations was relatively small, the score of the C-EIS was significantly associated with the TAS (*r* = 0.322, *P* < 0.01), which provides further evidence supporting the convergent validity. For the subscales, the details of convergent validity and discriminative validity are shown in Supplement 3.

## Discussion

The present study provides initial evidence for the reliability, construct validity, content validity, and convergent validity of the C-EIS in a sample of Chinese patients with cancer. The EIS is brief and may be completed within 10 min. The EIS is easy to perform because of the lack of complex sentences and calculations. Therefore, nurses and psychologists can use the EIS to assess the level of EI and the effect of emotional disclosure interventions.

The sample 2 group (*n* = 202) was recruited to evaluate the psychometric properties of the C-EIS. Based on the findings of the exploratory study, the C-EIS was modified to construct a Chinese version with four subscales: timidity, verbal inhibition, self-control, and disguise of feelings. The results of the factorial structure of the C-EIS of EFA were identical to the results of CFA. Factor analysis revealed four factors that fit easily into the four-dimensional model. This result is similar to the English version of the EIS, which also extracted four factors, although not every item in the principal component was the same. Item 9 (“Do you speak up for your rights?”) was problematic, with a low factor loading (<0.40), and item 11 (“Do you speak your mind even if it is bad for you?”) was deleted because of only one item in a different domain of content. The results from the validation sample indicate that the C-EIS had high validity, reliability, and a four-factor structure, which is the same as the original structure. The data provide further evidence of the multidimensional nature of EI. The shift of these items compared to the original EIS may be due to the potential differences in culture, samples, race, ethnicity, and/or some social factors between American and Chinese patients. The differences in the sample type of disease, sample size, age of participants, sex distribution, and disease condition between previous studies and the present study may result in differences in the items retained in the model.

The known-group comparison showed the construct validity to some extent. Two of the three hypotheses were confirmed, which means that the C-EIS distinguished between low and high known groups in patient disease characteristics that affected their expression of emotion. The expected hypothesis based on time since cancer diagnosis was not supported, which might indicate that patients with cancer adjust themselves and adapt to the cancer diagnosis and treatment (Peh et al., 2016). The results from the known-group comparison in this study showed that patients with higher stages of cancer had higher levels of EI, which reminds health-care professionals to pay more attention to patients with more malignant cancer.

Although the C-EIS score in this study was significantly associated with the TAS score, the association was relatively small, which is consistent with a previous study (Kessler et al., 2010; Grandi et al., 2011). Grandi et al. (2011) reported that EI was a concept similar to alexithymia. To some extent, alexithymia positively correlated with the subscale “verbal inhibition” in the EIS, although it was basically independent from the “self-control,” “timidity,” and “disguise of feelings” subscales in the EIS.

### Clinical Implications

The EIS might be useful in studies of the physiology of EI and influencing factors in patients with cancer. Of course, this simple assessment may be used in clinical practice with emotional disclosure intervention. The EIS identified high levels of EI in patients and assessed their response to the intervention. We also found that two variables affected EI levels of patients: cancer stage and type of cancer. The EIS serves as a reminder to health-care professionals to provide ongoing evaluation and intervention in patients with different types and stages of cancer.

### Limitations and Future Research

There are some limitations in this study. First, the current sample was relatively small. The patients from this study were limited to those with cancer at a tertiary grade A hospital in Guangzhou, so it is less persuasive to generalize these results to other populations and regions in China. Future studies should recruit more representative samples to replicate and verify the results in other populations and various regions of China and establish the Chinese norm of the C-EIS. It is also necessary to evaluate the minimum clinically important difference in future research before we use the EIS as a primary or secondary outcome in randomized controlled clinical trials (RCTs). Second, this study depended exclusively on the self-report data collection method, which may have some serious issues, such as bias or inaccurate reporting. Future research may include another objective approach to assess the influence of EI and its consequences, such as behavioral and physiological measures and experimental paradigms. Third, this validation study was based on the classic theory test (CTT), and it will be useful to perform item response theory (IRT) in future research to provide additional important information (Ye et al., 2019, 2020).

## Conclusions

The present study provided reliability and validity evidence of the C-EIS for assessing emotional characteristics in patients with cancer. Our findings confirmed four factors in the C-EIS, including verbal inhibition, self-control, disguise of feelings, and timidity. The C-EIS can be readily used to assess emotional care of patients with cancer in China, the level of EI, and the effect of emotional disclosure interventions.

## Data Availability Statement

The raw data supporting the conclusions of this article will be made available by the authors, without undue reservation.

## Ethics Statement

The studies involving human participants were reviewed and approved by the Medical Ethics Committee of Nanfang Hospital. Written informed consent to participate in this study was provided by the participants' legal guardian/next of kin.

## Author Contributions

LL collected and analyzed the data, interpreted the results, and wrote the manuscript. YX prepared the submission materials and collected the data. YW performed the analyses and revised the manuscript. XL designed the study and wrote the manuscript. CZ revised the manuscript critically. All authors contributed to the article and approved the submitted version.

## Conflict of Interest

The authors declare that the research was conducted in the absence of any commercial or financial relationships that could be construed as a potential conflict of interest.

## Publisher's Note

All claims expressed in this article are solely those of the authors and do not necessarily represent those of their affiliated organizations, or those of the publisher, the editors and the reviewers. Any product that may be evaluated in this article, or claim that may be made by its manufacturer, is not guaranteed or endorsed by the publisher.
