# Supplementary material for: Cross-Cultural Adaptation and Validation of the Emotional Inhibition Scale in a Chinese Cancer Sample
Source: Front Psychol. 2021 Sep 3;12:654777. doi: 10.3389/fpsyg.2021.654777 (PMC8446548; doi:10.3389/fpsyg.2021.654777)
Supplement: Supplementary file 1 [file Data_Sheet_1.pdf]

Supplement 1: Item Differential Functions Analysis Table

Category	Number	EIS (x±s)	t/F	P
Age			0.149	0.862[1]
18 ~ 44	62	5.61±2.40		
45 ~ 59	130	6.02±2.98		
>60	110	5.65±2.73		
Gender			2.631	**0.009**[2]
male	173	30.92±6.53		
female	129	29.69±6.11		
Religious belief			1.374	0.172[2]
No	68	31.01±6.15		
Yes	234	30.24±6.44		
Place of residence			2.366	**0.019**[2]
Town	156	31.28±5.93		
Countryside	146	29.48±6.72		
Marital status			1.028	0.313[2]
Married	279	30.48±6.42		
Single/divorced/widowed	23	29.57±5.94		
Education level			1.747	0.157[1]
Primary or under	69	30.46±6.00		
Junior high school	105	31.06±6.75		
Senior high school	58	30.96±6.02		
College or above	70	28.94±6.36		
Medical expenses payment method			1.701	0.184[1]
New rural medical insurance	118	30.90±46.6		
Social security	172	33.67±5.43		
Self-pay	12	6.92±3.03		
Household monthly income (Yuan)			0.948	0.389[1]
≤3,500	164	30.66±6.21		
3,500-5,000	97	30.52±6.50		
>5000	41	29.15±6.77		
Duration of disease (years)			0.890	0.447[1]
< 1 year	216	30.56±6.40		
2-3 years	57	29.32±6.01		
4-5 years	16	31.75±8.89		
> 5 years	13	31.15±3.132		
Types of cancer			2.147	**0.026**[1]
Lung cancer	56	31.55±6.57		
Breast cancer	56	29.77±5.89		
Prostate cancer	43	27.13±5.86		

Colon cancer	29	30.07±7.11		
Nasopharyngeal cancer	29	31.33±5.67		
Gastric cancer	23	31.83±5.87		
Liver cancer	19	29.78±5.49		
Rectal cancer	16	28.84±5.37		
Esophagus cancer	16	33.00±9.1		
Uterine cervical cancer	15	27.53±6.72		
Whether to transfer			1.164	0.246[2]
No	109	31.40±6.65		
Yes	192	29.91±6.16		
Whether recurrence			1.073	0.289[2]
No	39	31.21±6.78		
Yes	263	30.29±6.32		
Disease staging			4.470	**<0.01**[1]
I stage	10	24.30±5.33		
II stage	137	30.00±5.85		
III stage	45	30.36±6.72		
IV stage	110	31.50±6.66		
Recent treatment			1.645	0.148[1]
Surgery	107	30.05±6.31		
Chemotherapy	127	31.31±6.68		
Radiotherapy	8	30.75±6.925		
Targeted therapy	31	28.29±5.80		
Nutritional treatment	9	29.44±3.78		
Other	20	30.20±5.98		
Total	302	30.41±6.38		

[1] one way ANOVA；[2] t-test

Supplement 2: Real and Random Data Eigenvalues for N-Roots Based on Parallel Analysis

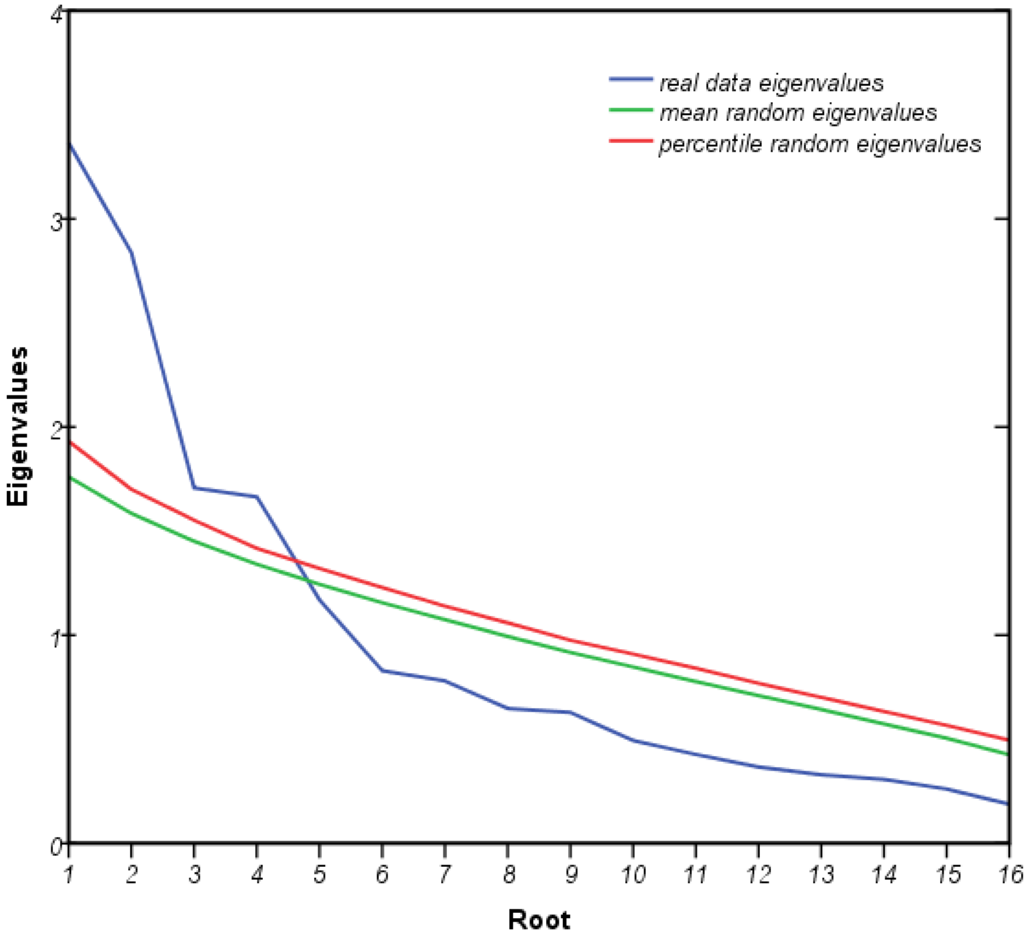

Supplement 3: Convergent validity and discriminative validity

	AVE	Verbal inhibition	Disguise of feeling	Self-control	Timidity
Verbal inhibition	0.777	**0.881**			
Disguise of feeling	0.596	.296	**0.772**		
Self-control	0.560	.298	.112	**0.748**	
Timidity	0.530	-.061	.208	.367	**0.728**

Supplement 4: The final version of the C-EIS

情绪抑制量表中文版

请阅读所有的问题，每个问题都有五个选项，根据问题选择最符合您情况的一个选项。 有些问题相似，但有一些细微的差别。 选项没有对错之分，请根据您的感受或行为进行选择。在回答问题前不要想太久，请尽快选出符合您情况的答案。

1.即使别人对你很粗鲁，你也会礼貌地对她（他）吗？

☐不会 ☐很少 ☐有时 ☐大多数时候 ☐总是

2.你是否觉得和别人谈论自己的感受是一件容易的事？

☐不会 ☐很少 ☐有时 ☐大多数时候 ☐总是

3.当你觉得自己被误解时，你是否觉得很难为自己辩护？

☐不会 ☐很少 ☐有时 ☐大多数时候 ☐总是

4.你会表达出你的感受吗？

☐不会 ☐很少 ☐有时 ☐大多数时候 ☐总是

5. 你会告诉别人你的真实想法吗？

□不会 □很少 □有时 □大多数时候 □总是

6. 你会不会觉得很难坚持自己的权利？

□不会 □很少 □有时 □大多数时候 □总是

7. 即使是亲密的朋友，你是否觉得很难和她（他）谈论你的真实感受？

□不会 □很少 □有时 □大多数时候 □总是

8. 当你生气时，你会努力控制自己的情绪吗？

□不会 □很少 □有时 □大多数时候 □总是

9.当你感到焦虑和担心时，你是否会努力表现得很平静？

□不会 □很少 □有时 □大多数时候 □总是

10.你是否会因为有些话可能会伤害他人而不说出来？

□不会 □很少 □有时 □大多数时候 □总是

11.你有没有觉得你被别人利用了？

□不会 □很少 □有时 □大多数时候 □总是

12.即使你感到难过的时候，你还会假装很开心吗？

☐不会 ☐很少 ☐有时 ☐大多数时候 ☐总是

13.你有没有过想告诉别人你的感受，最终却因为不好意思而没有说出口？

☐不会 ☐很少 ☐有时 ☐大多数时候 ☐总是

14.你会让你的朋友看出你的心情怎样吗？

☐不会 ☐很少 ☐有时 ☐大多数时候 ☐总是
